# The combination of excimer laser ablation and drug eluting balloon for treating superficial femoral arterial occlusion following stent implantation: A case report

**DOI:** 10.1097/MD.0000000000022935

**Published:** 2020-10-23

**Authors:** Xueliang Wu, Zhao Wang, Tian Li

**Affiliations:** a Department of Vascular and Glandular Surgery, The First Affiliated Hospital of Hebei North University, Zhangjiakou; b Department of General Surgery, Xingtai People's Hospital, Xingtai; c School of Basic Medicine, The Fourth Military Medical University, Xi’an, China.

**Keywords:** in-stent restenosis, excimer laser ablation, drug eluting balloon, superficial femoral arterial occlusion, following stent implantation

## Abstract

**Rationale:**

Recent research shows that in-stent restenosis (ISR) occurs in half of the patients treated with stenting of femoral and popliteal artery for lower extremity arteriosclerotic occlusive disease (LEASO). Combined therapy is mainly used clinically to obtain better medium- and long-term treatment outcomes and reduce the occurrences of reintervention, among which, the combination of excimer laser ablation (ELA) and drug eluting balloon (DEB) is a new and effective choice.

**Patient concerns:**

A 76-year-old male patient with ISR of right superficial femoral artery after stent implantation was reported.

**Diagnosis:**

Rechecking angiography indicated severe occlusion of the right superficial femoral artery. The physical examination showed that bilateral femoral and popliteal arteries were accessible whereas right dorsalis and posterior tibial arteries are unaccessible. Ankleolus brachial index (ABI) was 0.92 for left and 0.58 for right.

**Interventions:**

We performed the operation with ELA and drug balloon DEB on the right superficial femoral artery under local anesthesia and treated with oral antiplatelet drugs after operation.

**Outcomes:**

The combination treatment was very successful, and postoperative lower extremity arteriogram showed the blood flow was fluent and fast. No recurrence was discovered 3 months after the operation and he had no obvious symptom of claudication.

**Lessons:**

The combination of ELA and DEB is useful and effective for ISR of peripheral vessel after stent implantation, and this surgical method is worthwhile but need further clinical research for safety confirmation.

## 1 Introduction

ISR is commonly seen in patients with diabetic foot and more than 115,000 ISR patients need to receive secondary surgery annually. There are in-stent restenosis (ISR) in more than 50% of patients receiving femoral popliteal artery stent, which results from migration and proliferation of smooth muscle cells and formation of extracellular matrix resulting caused by plaque reconstruction and continuous compression of vascular intima. ISR, occurring commonly at long and complicated lesions with complex causes and mechanisms, is treated non-surgically and with vascular recanalization.^[1,2]^ Non-surgical treatment includes quitting smoking, control of blood sugar, blood lipids and blood pressure, anticoagulation, and active exercise, and medication. Vascular recanalization consists of conventional open surgery, Forgart catheter thrombectomy, intraductal contact thrombolysis, percutaneous transluminal angioplasty (PTA), percutaneous transluminal angioplasty and stenting (PTAS), and percutaneous plaque resection, excimer laser atherectomy (ELA), and endovascular brachytherapy (EVBT). Combined therapy is mainly used clinically to obtain better medium- and long-term treatment outcomes and reduce the occurrences of reintervention. In the present case, we presented a 76-year-old male patient with ISR of right superficial femoral artery after stent implantation, and aimed at reporting the experiences of ELA and DEM combination.

## 2 Case presentation

A 76-year-old male patient with Han Chinese was admitted on September 9, 2018, with intermittent claudication for 3 months and resting pain for 2 weeks. He received the stent implantation of right superficial femoral artery, and has symptoms of intermittent claudication of lower limbs, with claudication distance of 100 m ∼1 year ago. Angiography shows severe proximal and midcourse stenosis of the right superficial femoral artery and slight proximal stenosis of the left superficial femoral artery. He was treated with balloon dilatation and stent implantation of the right superficial femoral artery and recovered after the surgery, whereas it is relapsed with a claudication distance of 50 m 3 months ago. Rechecking angiography indicates severe occlusion of the right superficial femoral artery. He has a history of smoking, hypertension, and hyperlipidemia for 50, 25, and 10 years, respectively. The physical examination shows that bilateral femoral and popliteal arteries are accessible whereas right dorsalis and posterior tibial arteries are unaccessible. Ankleolus brachial index (ABI) is 0.92 for left and 0.58 for right.

**Figure 1:**
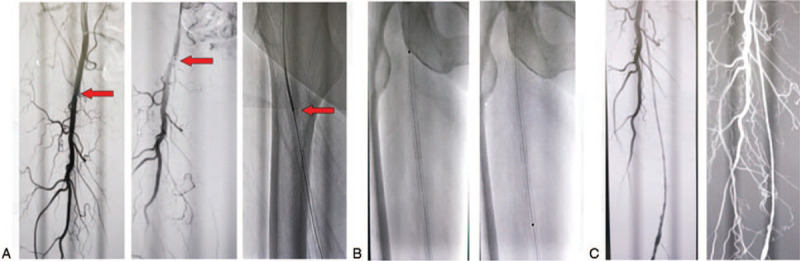
The implement of ELA. (A) Angiography shows ISR of right superficial femoral artery. (B) The thrombus is ablated by ELA. (C) This illustration shows the angiography after ablation.

**Figure 2:**
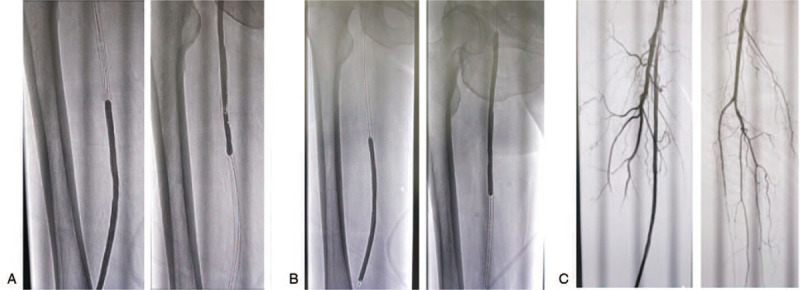
The implement of DEB. (A) Ordinary balloon dilatation. (B) Balloon dilatation by DEB. (C) Second angiography after DEB therapy.

He received excimer laser ablation (ELA) and drug balloon (DEB) on the right superficial femoral artery under local anesthesia on September 12, 2018. Surgical procedures include antidromic puncture of the left femoral artery with an arterial sheathing 6F canal. It was delivered into the right iliac artery, and digital subtraction angiography (DSA) shows a stent occlusion of the right superficial femoral artery (Fig. 1A). Then the long sheath was used to lead the V-18 guide wire and Turbo Elite Laser fiber conduit (Spectranetics Corporation, Colorado Springs) to go through the occlusion section. The tail of conducting wire is connected with CVX-300 laser generator and ablates plaque with an energy density of 30 mJ/mm^3^, 25 Hz, and 1 mm/s (Fig. 1B). The second DSA indicates the blood flow is restored (Fig. 1C) and the occlusion was treated with ordinary balloon (Fig. 2A) and DEB (Fig. 2B). The third DSA suggests that the blood flow is fluent and fast (Fig. 2C). The ischemic symptom was remitted and the ABI is 0.91. He was discharged successfully on September 15, 2018, and was ordered to take oral antiplatelet drugs. No recurrence was discovered 3 months after the operation and the blood flow of both lower limbs was fluent. And he had no obvious symptom of claudication, and vascular ultrasound showed a fluent blood flow.

## 3 Ethic statement

Being a Case Report with written consent, our institution does not require formal Ethical Approval. Written informed consent was obtained from the patient for publication of this case report.

## 4 Discussion

Nickel–titanium alloy stents has underwent a revolutionary change in the treatment of femoral popliteal artery diseases recently, whereas there are ISR in more than 50% of patients receiving femoral popliteal artery stent, which results from migration and proliferation of smooth muscle cells and formation of extracellular matrix resulting caused by plaque reconstruction and continuous compression of vascular intima.^[1,2]^ ISR is commonly seen in patients with diabetic foot and more than 115,000 ISR patients need to receive secondary surgery annually. ISR is traditionally treated with PTA combined with or without stent implantation (bare metal stent, stent graft, or drug-eluting stent).

**Figure 3:**
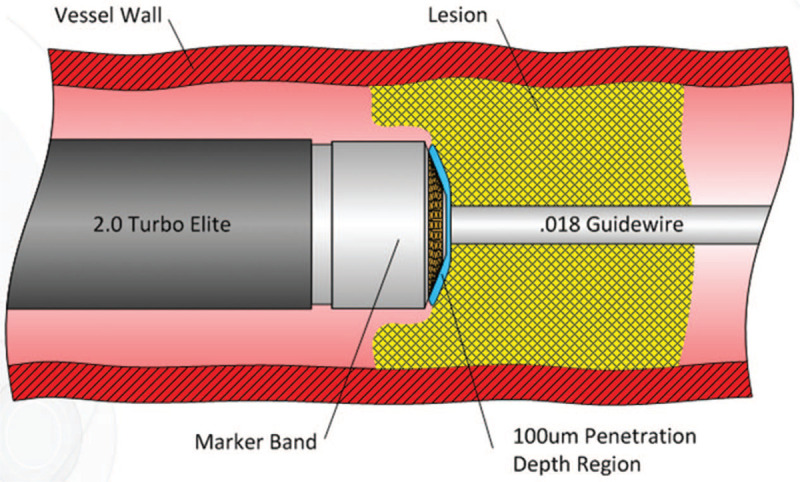
The design philosophy of ELA. This illustration shows the design philosophy of ELA.

**Figure 4:**
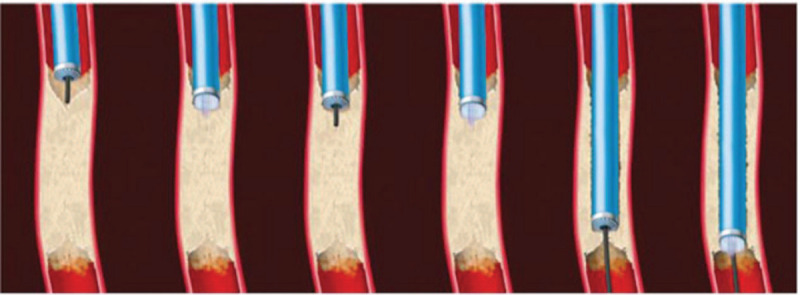
The detailed processes of ELA. In the step-by-step technique, the guidewire advances a few millimeters into the occlusion. The laser is activated while advancing the laser catheter, ablating the lesion until its tip is flush with the guidewire. The process is then repeated until the occlusion is crossed.

ELA has a huge advantage of ablating restenotic tissue and reducing/delaying the need for recurrent revascularization.^[3]^ ELA catheter consists of a large diameter of 0.1 mm fiber, catheter center of godet cavity, laser tube with V-18 or V-14 thread, thus ensuring the laser emission direction parallel to the blood vessels (Figs. 3 and 4).

Shammas and colleagues discovered that ELA has an favorable outcome in treating ISR of the femoropopliteal arteries.^[4]^ Laser ablation and penetration of an atretic pulmonary valve are feasible and safe. Moskowitz et al reported that the penetration of the aortic valve with the laser catheter enables subsequent introduction of various sizes balloon dilation. Notably, Dippel and colleagues enrolled 250 patients with chronic peripheral artery disease with femoropopliteal bare nitinol ISR and discovered that ELA with adjunctive PTA is superior than PTA alone. According to the fact that the effectiveness and safety of DEB in artery occlusion of lower limb,^[5]^ our study used the combination of ELA and DEB to treat the artery occlusion of the lower limb. And the patients received a good outcome. The present case further confirmed that the combination of ELA and DEB is useful and effective for ISR of peripheral vessel after stent implantation, and this surgical method is worthwhile but need further clinical research for safety confirmation.

## Author contributions

**Conceptualization:** Xueliang Wu, Tian Li.

**Data curation:** Xueliang Wu.

**Writing – original draft:** Zhao Wang.

**Writing – review & editing:** Xueliang Wu, Tian Li.
